# Targeted mutagenesis in soybean using the CRISPR-Cas9 system

**DOI:** 10.1038/srep10342

**Published:** 2015-05-29

**Authors:** Xianjun Sun, Zheng Hu, Rui Chen, Qiyang Jiang, Guohua Song, Hui Zhang, Yajun Xi

**Affiliations:** 1 College of Agronomy, Northwest A&F University, Yangling, Shaanxi 712100, China; 2 National Key Facilities for Crop Genetic Resources and Improvement, Institute of Crop Sciences, Chinese Academy of Agricultural Sciences, Beijing 100081, China; 3 Tianjin Institute of Agricultural Quality Standard and Testing Technology, Tianjin Academy of Agricultural Sciences, Tianjin 300381, China

## Abstract

Genome editing is a valuable technique for gene function analysis and crop improvement. Over the past two years, the CRISPR-Cas9 system has emerged as a powerful tool for precisely targeted gene editing. In this study, we predicted 11 *U6* genes in soybean (*Glycine max* L.). We then constructed two vectors (pCas9-GmU6-sgRNA and pCas9-AtU6-sgRNA) using the soybean *U6-10* and Arabidopsis *U6-26* promoters, respectively, to produce synthetic guide RNAs (sgRNAs) for targeted gene mutagenesis. Three genes, *Glyma06g14180*, *Glyma08g02290* and *Glyma12g37050,* were selected as targets. Mutations of these three genes were detected in soybean protoplasts. The vectors were then transformed into soybean hairy roots by *Agrobacterium rhizogenes* infection, resulting in efficient target gene editing. Mutation efficiencies ranged from 3.2–9.7% using the pCas9-AtU6-sgRNA vector and 14.7–20.2% with the pCas9-GmU6-sgRNA vector. Biallelic mutations in *Glyma06g14180* and *Glyma08g02290* were detected in transgenic hairy roots. Off-target activities associated with *Glyma06g14180* and *Glyma12g37050* were also detected. Off-target activity would improve mutation efficiency for the construction of a saturated gene mutation library in soybean. Targeted mutagenesis using the CRISPR-Cas9 system should advance soybean functional genomic research, especially that of genes involved in the roots and nodules.

Genome editing is an important tool for gene function analysis, gene therapy and crop improvement. In recent years, three genome editing techniques have been developed that rely on zinc-finger nucleases (ZFNs), transcription activator-like nucleases (TALENs) and clustered regularly interspaced short palindromic repeat (CRISPR)/CRISPR-associated protein (Cas) system^1^. All three methods induce double-stranded breaks (DSBs) in the target genome DNA, which are subsequently repaired through non-homologous end joining (NHEJ) and homologous recombination (HR)^2^. ZFNs and TALENs target the genome through protein-DNA interactions, whereas genomic DNA editing by the CRISPR-Cas system is based on short RNA-DNA base pairing^1^,^3^,^4^. Compared with ZFNs and TALENs, genome editing with the CRISPR-Cas system is simpler, faster and more efficient. The CRISPR-Cas system has consequently been widely applied to genome editing in eukaryotic cells during the past two years^1^.

The CRISPR-Cas system, an adaptive immunity system against foreign nucleic acid invaders in prokaryotes, exists in most archaea and numerous bacteria^5^. The system has been categorized into three types (I, II, and III) based on the *Cas* genes and CRISPR sequences present^6^,^7^,^8^. Types I and III contain multiple CAS proteins that form a complex for degrading foreign DNA/RNA. Type II directs the cleavage of targeted foreign DNA using a single CAS9 protein, which makes it the system of choice for targeted genome engineering. In the Type-II CRISPR-Cas9 system, CRISPR RNA (crRNA) hybridizes with a small trans-activating CRISPR RNA (trancrRNA) to form mature dual crRNA^9^,^10^. The mature crRNA combines with Cas9 to form a functional complex. When the complex recognizes a short seed sequence in the vicinity of a typical 5’-NGG-3’ protospacer-adjacent motif (PAM) by RNA/DNA base pairing, Cas9 cleaves the target DNA^11^,^12^,^13^. Mature crRNA containing trancrRNA and crRNA can be replaced in the laboratory with a single synthetic guide RNA (sgRNA)^10^. Consequently, only sgRNA and Cas9 protein are needed to make genome editing simple and efficient. The CRISPR-Cas9 system has been widely applied in genetic studies of prokaryotes and eukaryotes over the past two years^14^.

In plants, the CRISPR-Cas9 system has been successfully used in various species including *Arabidopsis thaliana*, *Nicotiana benthamiana*, rice, tobacco, sorghum, wheat, maize, orange and liverwort^15^,^16^,^17^,^18^,^19^,^20^,^21^,^22^. In these applications, *Cas9* is expressed by the Cauliflower mosaic virus (CaMV) 35s promoter or a gene-specific promoter. A nuclear localization signal (NLS) sequence is fused to the *Cas9* gene, which delivers CAS9 to the genomic nuclei. To design the sgRNA, the 20-bp sequence following the PAM in the target DNA is selected as the sgRNA seed. These 20-bp sgRNA seed regions are abundant in plant genomes^16^, with more than 90% of rice genes containing a specific sgRNA seed^23^. Several software programs have been developed to identify target-specific sgRNA seeds^24^,^25^,^26^. RNA polymerase-III promoters such as *U6* and *U3* are typically used to express the sgRNA^27^. These polymerase-III promoters express transcripts with a purine initiated at the first nucleotide. The purine may or may not affect base pairing between the sgRNA and the target DNA. The CaMV 35s promoter is also used to express the sgRNA without an additional nucleotide^21^. Several different sgRNAs can be co-expressed within a single CRISPR-Cas9 system to target multiple DNA sites simultaneously. In plants, DSBs induced by CRISPR-Cas9 can be repaired by HR or NHEJ, the latter primarily responsible for genomic insertion or deletion (indel) mutations. The CRISPR-Cas9 system is a highly efficient tool to obtain targeted mutant transgenic plants with a high frequency of mutation^27^. Biallelic mutations have also been observed at high frequencies in T_0_ transgenic plants^16^,^19^. Mutations can be stably inherited in the next generation^28^,^29^,^30^. The CRISPR-Cas9 system is a powerful tool for the advancement of function genomics research. Targeted mutagenesis has also been applied to crop improvement. In wheat, *TaMLO* mutants induced by the CRISPR-Cas9 system have shown broad-spectrum resistance to powdery mildew^31^.

Mutagenesis has played an important role in functional genomics over the past two decades. Targeted mutagenesis is an efficient tool for functional genomics research. Although ZFNs have been used in recent years for targeted mutagenesis in soybean (*Glycine max* L.)^32^, construction difficulties, high cost and modest efficacy limit their application^1^. In this study, we used the CRISPR-Cas9 system to efficiently perform targeted mutagenesis in soybean protoplasts and hairy roots. Targeted mutagenesis using the CRISPR-Cas9 system can advance soybean functional genomic research, especially that of genes involved in roots and nodules.

## Results

### Prediction of *U6* promoters in soybean

**Figure 1:**
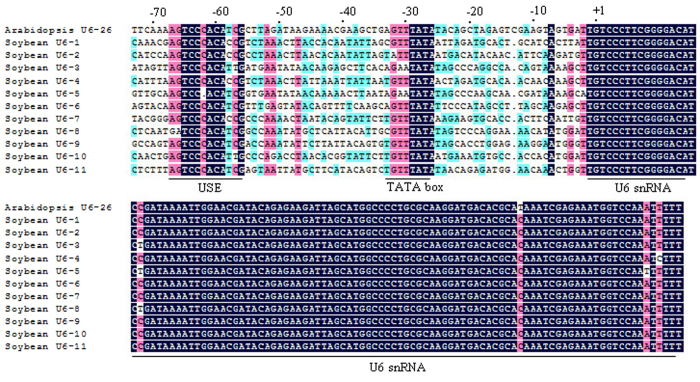
Alignment of 11 soybean *U6* and Arabidopsis *U6-26* genes. Soybean *U6* and Arabidopsis *U6-26* genes are highly conserved. Upstream sequence element (USE), TATA-box and *U6* small nuclear RNA (snRNA) sequence regions are underlined.

The *U6* promoter is typically used to drive the expression of sgRNA in various plants^27^. The Arabidopsis *U6-26* promoter has been used to generate sgRNA in Arabidopsis and *N*. *benthamiana*; the rice *U6* promoter has been used in rice and sorghum. By comparison with the Arabidopsis *U6* small nuclear RNA (snRNA) sequence, we predicted 11 *U6* genes in the soybean genome. These 11 *U6* genes were distributed on seven chromosomes, with 2 *U6* genes (*U6-7* and *U6-8*) clustered on a 6.1-kb fragment on chromosome 16 (Supplementary Table S1). Plant *U6* promoters contain the following two conserved elements: an upstream sequence element (USE; consensus sequence RTCCCACATCG) and a TATA-like box^33^. These two elements, separated by a suitable distance, are necessary for *U6* gene transcription^33^. Promoter sequences of the 11 *U6* genes were extracted from the soybean genome. Multiple sequence alignments revealed that the *U6-5* promoter had a C-nucleotide deletion in the USE, whereas the other 10 *U6* promoters contained both conserved elements (Fig. 1). The USE and TATA-like-box conserved sequences in soybean are RTCCCACA(T/C)(T/C)G and GTTTATA, respectively. The presence of these conserved elements suggests that the 10 soybean *U6* promoters may have the transcriptional activity to generate sgRNAs in soybean.

### Evaluation of the CRISPR-Cas9 system for gene editing in soybean

**Figure 2:**
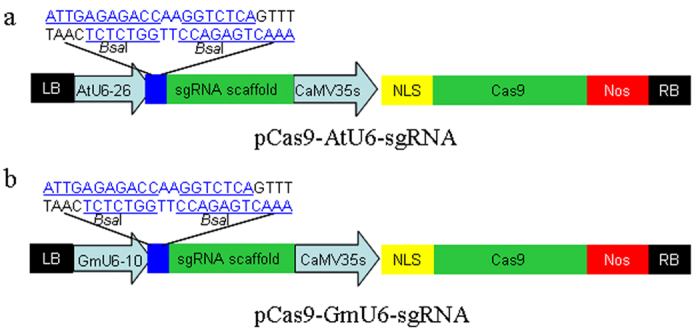
Construction of binary vectors for genome editing in soybean. Cas9 fused with a single nuclear localization signal (NLS) is expressed with a Cauliflower mosaic virus 35s (CaMV 35s) promoter. Synthetic guide RNA (sgRNA) is derived using U6 promoters. (**a**) *Arabidopsis thaliana U6-26* promoter (**b**) *Glycine max U6-10* promoter. Sequences containing two *Bsa*I sites are located between the *U6* promoter and the sgRNA scaffold. These sequences can be easily replaced with a gene-specific sgRNA seed. LB: left border; RB: right border.

We constructed two binary vectors to express sgRNAs and *Cas9* for gene editing (Fig. 2). In both vectors, the CaMV 35s promoter was used to drive the expression of *Cas9*. Two RNA polymerase-III (Pol III) promoters, *AtU6-26* and *GmU6-10*, were selected to generate sgRNAs in the two vectors (pCas9-AtU6-sgRNA and pCas9-GmU6-sgRNA, respectively). Two *Bsa*I sequences, easily replaceable by 20-bp sgRNA seed sequences, were introduced between the *U6* promoter and the sgRNA scaffold in the vectors.

To detect the activity of these two vectors in soybean, we selected three genes (*Glyma06g14180*, *Glyma08g02290* and *Glyma12g37050*) as targets for gene editing in soybean. For each gene, we designed a different sgRNA seed with a restriction site in the vicinity of the PAM (Supplementary Table S2). A total of six binary vectors were therefore generated to evaluate targeted mutagenesis in this study.

### Targeted mutagenesis in soybean protoplasts

**Figure 3:**
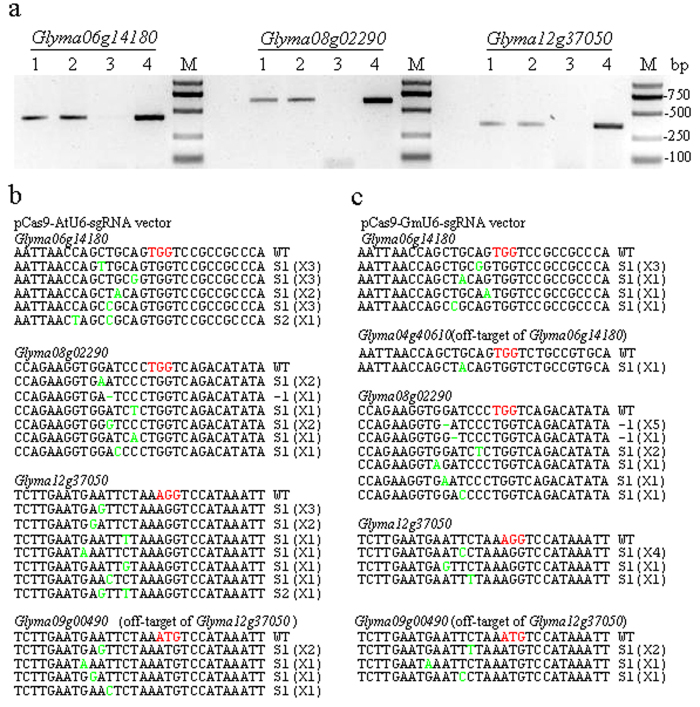
Targeted mutagenesis in soybean protoplasts. (**a**) Detection of mutations using restriction enzyme-PCR (RE-PCR). Lanes 1 and 2: PCR products of digested genomic DNA from protoplasts treated with pCas9-AtU6-sgRNA and pCas9-GmU6-sgRNA, respectively; Lanes 3 and 4: PCR products of digested and undigested genomic DNA, respectively, from wild-type controls. (**b**) and (**c**) Sequence-based detection of mutations induced by pCas9-AtU6-sgRNA and pCas9-GmU6-sgRNA vectors, respectively. Wild-type sequences of the target genes and off-target genes are shown with the protospacer-adjacent motif sequence highlighted in red. The change in the number of nucleotides is shown to the right of each sequence. D: deletion; S: substitution. Nucleotide substitutions are shown in green. The number of clones for each mutant is given in brackets.

We first verified the activity of the CRISPR-Cas9 system in soybean protoplasts. The vectors were transformed into soybean protoplasts using the polyethylene glycol (PEG)-mediated transformation method. After 48 h of incubation in darkness at room temperature, the transformed protoplasts were collected for genomic DNA extraction. A restriction enzyme PCR (RE-PCR) assay was used to detect mutations in the targeted genes. The genomic DNAs for various targeted mutant genes were completely digested with restriction enzymes. The mutant genes were not digested as they lost the enzyme sites and could be amplified using the gene-specific primers. The PCR results confirmed that all six vectors were able to induce targeted gene mutations (Fig. 3a). Sequence analysis revealed that nucleotide substitutions had occurred in *Glyma06g14180* and *Glyma12g37050* (Figs. 3b,c), suggesting that the DSBs in these two genes were repaired by the HR pathway in the soybean protoplasts. One nucleotide deletion and one substitution were found in *Glyma08g02290* (Figs. 3b,c). The DSBs of *Glyma08g02290* were repaired through both the HR and NHEJ pathways in soybean protoplasts.

### Targeted mutagenesis in soybean hairy roots

**Figure 4:**
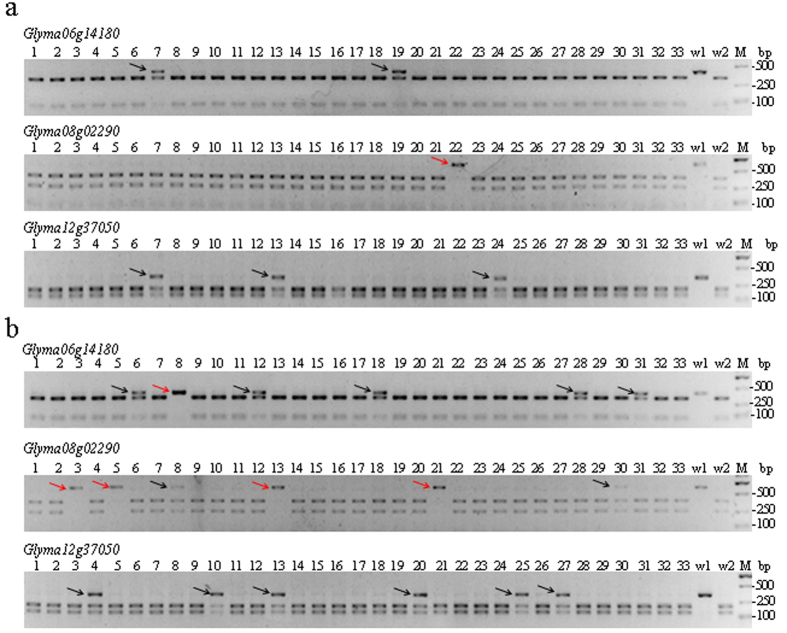
Detection of mutants using the PCR-restriction enzyme (PCR-RE) assay. Detection of mutations using the PCR-restriction enzyme (PCR-RE) assay. Lanes 1–33: the digested DNA of the PCR products amplified from the independent hairy root samples; The monoallelic and biallelic mutants are shown with black and red arrows, respectively. w1 and w2: the undigested and digested DNA, respectively, from the PCR products amplified from wild-type controls. (**a**) Targeted mutations induced by the pCas9-AtU6-sgRNA vector. (**b**) Targeted mutations induced by the pCas9-GmU6-sgRNA vector.

*Agrobacterium rhizogenes* (*A. rhizogenes*)-mediated transformation is a rapid, efficient, simple and inexpensive method for the studying soybean root biology^34^. To detect the targeted gene mutations in soybean roots, we introduced the six binary vectors into *A. rhizogenes* strain K599 and then infected soybean seedling hypocotyls to induce hairy roots. Genomic DNA was collected and extracted for further detection of the target gene mutations from the hairy roots for each of the six vectors. Soybean is a diploid plant and genes have two copies in the homologous chromosomes. The target gene induced by the CRISPR-Cas9 system has three types in the hairy roots. Type I is no mutation of the target gene. Type II is a monoallelic mutation where one gene is mutated and the other allelic gene is no mutated. Type III is a biallelic mutation where both of the two allelic genes are mutated (Supplementary Figure S1). The gene is amplified using gene specific primers and then digested completely with the restriction enzyme (PCR-RE assay). When the gene mutation is induced by the CRISPR/Cas9 system, the restriction enzyme site in the gene is destroyed. The results of PCR-RE assay for the non-mutation show two digested bands. For the monoallelic mutation, the results are three bands with one undigested band from the mutated gene and two digested bands from non-mutated allelic gene. For the biallelic mutation, both of the two allelic genes are mutated and the PCR-RE assay shows only a single undigested band (Supplementary Figure S1). The PCR-RE assay shows that gene mutations were induced using all six vectors (Fig. 4 and Supplementary Figures S2–S7). The undigested bands from the PCR-RE assay were cloned and sequenced to confirm the mutations. Sequence analysis indicated that the types of mutations differed between the genes (Supplementary Figures S2–S7). Most *Glyma06g14180* mutations were single nucleotide insertions, whereas the majority of the detected mutations in *Glyma08g02290* and *Glyma12g37050* involved multiple-nucleotide deletions. Although rare in the soybean hairy roots, nucleotide substitutions were the major type of mutation induced in soybean protoplasts using the CRISPR-Cas9 system. Mutation efficiencies differed between the pCas9-GmU6-sgRNA and pCas9-AtU6-sgRNA vectors (Table 1), with markedly higher efficiencies obtained with all three genes using the pCas9-GmU6-sgRNA vector. Mutation efficiencies with the pCas9-GmU6-sgRNA vector for *Glyma06g14180*, *Glyma08g02290* and *Glyma12g37050* were 14.7, 20.2 and 17.9%, respectively, with corresponding efficiencies of 6.6, 3.2 and 9.7% using the pCas9-AtU6-sgRNA vector.

**Figure 5:**
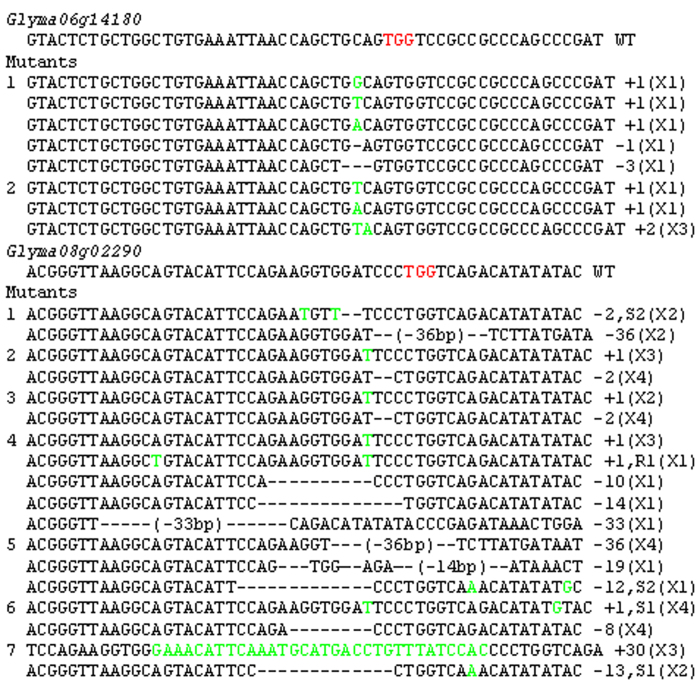
Gene sequences from 9 independent biallelic mutants. Gene sequences are shown for 9 independent biallelic mutants. Wild-type sequences of the target genes are shown with the protospacer-adjacent motif sequence highlighted in red. The change in the number of nucleotides is shown to the right of each sequence. +: insertion; D: deletion; S: substitution. Inserted and substituted nucleotides are shown in green.

Biallelic mutations can be detected in T_0_ transgenic plants using the CRISPR-Cas9 system^16^,^19^. We detected several biallelic mutations of *Glyma06g14180* and *Glyma08g02290* using the PCR-RE assay (Supplementary Figures S3–S5). A higher frequency of biallelic mutants was observed in *Glyma08g02290*. Twelve of 19 *Glyma08g02290* mutants generated using the pCas9-GmU6-sgRNA vector and 2 of 3 *Glyma08g02290* mutants induced by the pCas9-AtU6-sgRNA vector were biallelic (Table 1). Sequencing of several gene clones from independent biallelic mutant roots revealed a variety of mutations per root (Fig. 5), which suggests that the CRISPR-Cas9 system continued to modify the genes during hairy root development.

### Off-target activity in soybean

The CRISPR-Cas9 system can tolerate several mismatches between the sgRNA seed and its target, especially in the first 12 nucleotides at the 5’ end of the sgRNA seed^35^,^36^,^37^, which suggests that off-target activity is common with the CRISPR-Cas9 system. We accordingly searched the soybean genome for homologs of the three targeted genes in this study. We found that *Glyma06g14180* and *Glyma04g40610* had the same target sequence and that the sequences of *Glyma08g02290* and *Glyma05g37270* were also identical to one another. *Glyma12g37050* and *Glyma09g00490* differed by a single nucleotide at the PAM site (AGG vs. ATG). Mutations in *Glyma04g40610* and *Glyma09g00490* induced by the CRISPR-CAS9 system using primers for *Glyma06g14180* and *Glyma12g37050* were detected in protoplasts and hairy roots (Fig. 3 and Supplementary Figures S3,S6 and S7).

## Discussion

In this study, we used two *U6* promoters, Arabidopsis *U6-26* and soybean *U6-10*, to generate sgRNA. Mutation efficiencies in the three target genes were significantly increased by the use of the soybean *U6-10* promoter (Table 1), which may be related to the *U6* promoter activity. The transcriptional efficiency of the different *U6* promoters varies in Arabidopsis^38^. Eleven *U6* promoters were predicted in soybean, which provided the opportunity to select a suitable *U6* promoter for the expression of sgRNA in soybean. The choice of promoter is critical, as high concentrations of the Cas9-sgRNA complex can increase off-target activity^35^,^37^.

Mutagenesis is a powerful tool for the studying gene function. The mutations induced by T-DNA insertion, chemical agents and physical treatments are random, which make it difficult to obtain the target mutants. Targeted mutagenesis technologies, such as TALEN, ZFN and CRISPR-Cas9 approaches, are powerful tools to generate target gene mutations. Compared with TALENs and ZFNs, the CRISPR-Cas9 system efficiently produces mutations and is easy to use^1^. In this study, we successfully used the CRISPR-Cas9 system for target gene mutation in soybean. The mutation efficiencies are ranged from 14.7% to 20.2% (Table 1). Sequencing of several gene clones from the mutant roots revealed that the CRISPR-Cas9 system continued to modify the genes during hairy root development, which suggests that the mutation efficiency would be increased given enough time for the development of the transgenic plants. The high efficiency of the target gene mutation can improve the research on gene function in soybean.

Biallelic mutations can be detected and their phenotypes observed in T_0_ transgenic plants using the CRISPR-Cas9 system^16^,^19^. In a study by Ron *et al.*^39^, the CRISPR-Cas9 system mediated by *A. rhizogenes* was used to produce a targeted mutation in the *SHORT-ROOT* (*SHR*) gene in tomato transgenic hairy roots. The phenotype of the resulting mutant was consistent with Arabidopsis *shr* mutants. In our study, biallelic mutations in *Glyma06g14180* and *Glyma08g02290* were detected in transgenic hairy roots (Fig. 5 and Supplementary Figures S3-S5). *Glyma08g02290* had a higher number of biallelic mutations, with 12 of 19 root samples showing mutations (Table 1). Biallelic mutants can be detected easily using the PCR-RE assay (Supplementary Figures S3-S5). Biallelic mutants are the ideal materials for researching gene function. Compared to the inefficient and time-consuming transformation mediated by *Agrobacterium tumefaciens* (*A. tumefaciens*), transformation mediated by *A. rhizogenes* is easy, quick and efficient in soybean^34^. Transgenic hairy roots can be obtained within one month with transformation efficiencies up to 80%. A large number of genes involved in the roots and nodules have been identified in soybean by next-generation sequencing^40^. It would be easy to generate the target gene mutants using the CRISPR-Cas9 system mediated by *A. rhizogenes*, which would lead to advances in soybean root biology research.

Off-target activity is common using the CRISPR-Cas9 system. In our study, we detected off-target gene mutations for *Glyma06g14180* and *Glyma12g37050* (Fig. 3 and Supplementary Figures S3, S6 and S7). Off-target activity limits the application of the CRISPR-Cas9 system, but several methods are available to reduce this impediment. Decreasing sgRNA-Cas9 concentrations can increase on-target specificity *in vitro*^35^,^37^. Off-target activity can be reduced 50- to 1500-fold using double-nicking mediated by a *Cas9* nickase mutant (*Cas9n*)^41^. Use of truncated gRNAs (tru-gRNAs), a shorter sgRNA seed (typically 17 or 18 nucleotides) complementary to the target, can also decrease off-target activity by 5000-fold or more^42^. Although these methods can effectively reduce off-target activity, the best strategy is identification of gene-specific sgRNA seeds. Fortunately, 97.3% of annotated transcription units (TUs) have specific sgRNA seeds in soybean; these TU-specific sgRNA seeds can be identified by searching the CRISPR-PLANT database (http://www.genome.arizona.edu/crispr)^43^.

**Figure 6:**
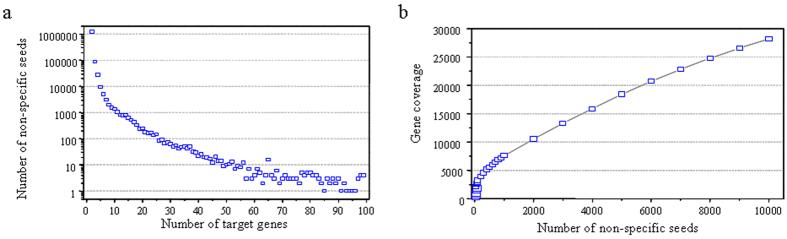
Non-specific synthetic guide RNA (sgRNA) seeds in soybean. (6a) Distribution of non-specific sgRNA seeds and the number of their target genes. More than 1 million sgRNA seeds were associated with two target genes; approximately 100,000 sgRNA seeds were able to target three genes. sgRNA seeds having more than 100 target genes are not shown. (6b) Maximal gene coverage of non-specific sgRNA seeds. The non-specific sgRNA seeds were sorted by their target gene numbers before calculating the maximal gene coverage.

Some mutation libraries have been developed by chemical agents and physical treatments in soybean^44^,^45^,^46^, but the mutants induced by these treatments are random and complex. T-DNA-induced mutagenesis has been widely applied in model plants such as Arabidopsis and rice^47^,^48^. Successful T-DNA insertion mainly depends on efficient of *A. tumefaciens*-mediated transformation. In soybean, the creation of large numbers of mutants using T-DNA insertion is not feasible, as transformation efficiency mediated by *A. tumefaciens* is low in this species. Nevertheless, the acquisition of target mutants is still time-consuming and inefficient because T-DNA-based mutagenesis is random. Off-target activity can be exploited for the construction of a saturated gene mutation library in soybean. The CRISPR-Cas9 system can tolerate several mismatches between the sgRNA seed and its target, especially in the first 12 nucleotides at the 5’ end of the sgRNA seed^35^,^36^,^49^. With respect to these 12 nucleotides, sgRNA seeds having fewer than four mismatches with other sequences in our study were considered to be non-specific sgRNA seeds. A total of 13,103,481 sgRNA seeds were predicted in soybean genes, of which 5,631,730 were specific and 7,469,546 were non-specific (Supplementary Figure S8). The number of specific sgRNA seeds as well as their coverage (99.5% of soybean genes) is consistent with results obtained by Xie *et al*.^43^. The huge quantity of non-specific sgRNA seeds allows the targeting of two or more genes in one transformation in soybean (Fig. 6a). Off-target activity produces numerous mutations covering different genes in T_0_ transgenic soybeans. The resulting mutants can be segregated to produce unique mutations in the progeny, which, similar to the application of Ac/Ds transposons or Tnt1 retrotransposons in T-DNA transformations, improves mutation efficiency^50^,^51^. In our study, the seeds of *Glyma06g14180* or *Glyma12g37050* were detected to produce two gene mutations (*Glyma06g14180* and *Glyma04g40610*, *Glyma12g37050* and *Glyma09g00490*) respectively in one transgenic plant (Figure S3, S6 and S7). By exploiting off-target activity, the number of transgenic soybean plants required to produce a saturated mutation library can be reduced dramatically (Fig. 6b).

## Methods

### Plant material

The soybean cultivar Williams 82 was used in this study. In preparation for *A. rhizogenes*-mediated transformation, seeds were sterilized for 7 h with chlorine gas. Seeds were germinated under 16-h light/8-h dark at 25 °C in a humidity chamber. After one week, healthy plants were selected for transformation. To generate protoplasts, seeds were germinated under 16-h light/8-h dark at 25 °C in a low-humidity chamber. Fresh leaves were collected for protoplast preparation from 2-week-old seedlings.

### Vector construction

A codon-optimized *cas9* gene with a NLS was obtained from Professor Qu (Qu, State Key Laboratory for Protein and Plant Gene Research, Peking-Tsinghua Center for Life Sciences, College of Life Sciences, Peking University). The *cas9* gene was amplified by phusion polymerase (NEB, Massachusetts, USA) using *cas9*-specific primers (Supplementary Table S3) and cloned into pCambia3301 vector by replacing of the *gus* gene.

Arabidopsis *U6-26* and soybean *U6-10* promoters with sgRNA were synthesized (Genscript, Nanjing, China) (Supplementary Figure S9 and S10) and cloned into pUC57-Kan vectors to generate pUC57-AtU6-26-sgRNA and pUC57-GmU6-10-sgRNA plasmids, respectively. These two plasmids were digested completely using *Bsa*I (NEB, Massachusetts, USA) and purified with a TIANquick Midi purification kit (Tiangen, Beijing, China). Three target gene oligonucleotides (Supplementary Table S2) were annealed to form sgRNA seeds and were then ligated into the pUC57-AtU6-26-sgRNA and pUC57-GmU6-10-sgRNA vectors. These six vectors and the pCambia3301-Cas9 vector were digested completely using *Eco*RI and *Hind*III. After digestion, the pCambia3301-Cas9 vector and AtU6-26-sgRNAs and GmU6-10-sgRNAs of different genes were purified with a TIANgel Midi purification kit (Tiangen, Beijing, China) and ligated overnight using T4 DNA ligase (Fermentas) to obtain pCas9-AtU6-sgRNA and pCas9-GmU6-sgRNA vectors for different target genes.

### Protoplast isolation and transformation

Soybean protoplasts were prepared from fresh leaves as described by Yoo *et al.*^52^ with some modifications. Briefly, 20 fresh leaves were cut into small strips and immediately transferred into 10 ml digestion solution (0.5% cellulose R10, 0.5% macerozyme R10, 0.1% pectolase Y23, 0.6 M mannitol, 10 mM 4-morpholineethanesulfonic acid (MES) pH 5.7, 20 mM KCl, 10 mM CaCl_2_ and 0.1% BSA). The leaf strips were vacuum infiltrated for 30 min in the dark using a vacuum pump at −15 to −20 mm Hg and digested for 6 h with agitation at 30 rpm. The other steps are followed as described by Yoo *et al.*^52^. The protoplasts were re-suspended in MGG solution (4 mM MES pH 5.7, 0.4 M mannitol, 15 mM MgCl_2_) for the plasmid transformation. Plasmids were transformed into protoplasts mediated by PEG as described by Yoo *et al.*^52^.

### Transformation mediated by *A. rhizogenes*

The binary vectors were transformed into soybean by *A. rhizogenes* as described by Kereszt *et al.*^34^.

### Detection of mutations in target genes

Genomic DNA was extracted using a DNAquick Plant System (Tiangen, Beijing, China) according to the manufacturer’s protocol with a minor modification: genomic DNA from soybean hairy roots was precipitated using Dr.GenTLE Precipitation Carrier (Takara, Dalian, China). To detect mutations in soybean protoplasts, the genomic DNA was digested with restriction enzyme (*Pst*I, *Bam*HI and *Eco*RI for mutant detection of *Glyma06g14180*, *Glyma08g02290* and *Glyma12g37050* respectively). After digestion, the target genes were amplified with gene-specific primers, and the PCR fragments were ligated to an p*EASY*-T1 vector (Transgen, Beijing, China) for sequencing. To detect mutations in hairy roots, the target genes were amplified by PCR using gene-specific primers (Supplementary Table S3). The PCR products were purified using TIANquick N96 Purification kit (Transgen, Beijing, China) and digested for three hours with *Pst*I, *Bam*HI and *Eco*RI, respectively. The undigested bands were purified using a TIANgel Midi purification kit (Tiangen, Beijing, China) and then ligated to a p*EASY*-T1 vector (Transgen, Beijing, China). Several clones were randomly selected and sequenced to detect gene mutations.

### Bioinformatic analysis

Soybean genome and annotation data were downloaded from the plantGDB database (http://www.plantgdb.org/). The bioinformatic analysis pipeline was primarily constructed using customized Perl scripts and the USEARCH program^53^. For specificity assessment of sgRNA seeds, 20-nt long sgRNA spacer sequences adjacent to NGG PAM sites were excluded from both strands of the soybean chromosome sequences. For specificity analysis, sgRNA seeds were first grouped according to the identity of the eight nucleotides at the 3–’, end. The first 12 nucleotides at the 5–’, end were then compared among members of the same group. sgRNA seeds with no less than four mismatches were regarded as specific candidates; the remaining seeds, including repeat sequences, were considered to be non-specific.

## Additional Information

**How to cite this article**: Sun, X. *et al.* Targeted mutagenesis in soybean using the CRISPR-Cas9 system. *Sci. Rep.* **5**, 10342; doi: 10.1038/srep10342 (2015).

## Supplementary Material

Suplementary Information

## Figures and Tables

**Table 1:** Gene mutations in three target genes using different vectors.

**Target gene**	**pCas9-GmU6-sgRNA vector**				**pCas9-AtU6-sgRNA vector**			
	**examined hairy roots**	**monoallelic mutants**	**biallelic mutants**	**Mutation efficiency**	**examined hairy roots**	**monoallelic mutants**	**biallelic mutants**	**Mutation efficiency**
*Glyma06g14180*	95	12	2	14.7%	91	6	0	6.6%
*Glyma08g02290*	94	7	12	20.2%	93	1	2	3.2%
*Glyma12g37050*	84	15	0	17.9%	72	7	0	9.7%
