# Supplementary material for: Targeted mutagenesis in soybean using the CRISPR-Cas9 system
Source: Sci Rep. 2015 May 29;5:10342. doi: 10.1038/srep10342 (PMC4448504; doi:10.1038/srep10342)
Supplement: Suplementary Information [file srep10342-s1.pdf]

Supplementary Information

Targeted mutagenesis in soybean using the CRISPR-Cas9 system

Zheng Hu[1,*,#], Xianjun Sun[1,2,#], Rui Chen[3], Qiyang Jiang[1], Guohua Song[1], Hui Zhang[1] and Yajun Xi[2,*]

[1] National Key Facilities for Crop Genetic Resources and Improvement, Institute of Crop Sciences, Chinese Academy of Agricultural Sciences, Beijing 100081, China

[2] College of Agronomy, Northwest A&F University, Yangling, Shaanxi 712100, China

[3] Tianjin Institute of Agricultural Quality Standard and Testing Technology, Tianjin Academy of Agricultural Sciences, Tianjin 300381, China

*Authors for correspondence. ZH: huzheng@caas.cn; YX: xiyajun2002@126.com.
# These authors contributed equally to this work.

Supplementary Information contains:
Supplementary Figures S1-S10
Supplementary Tables S1-S3

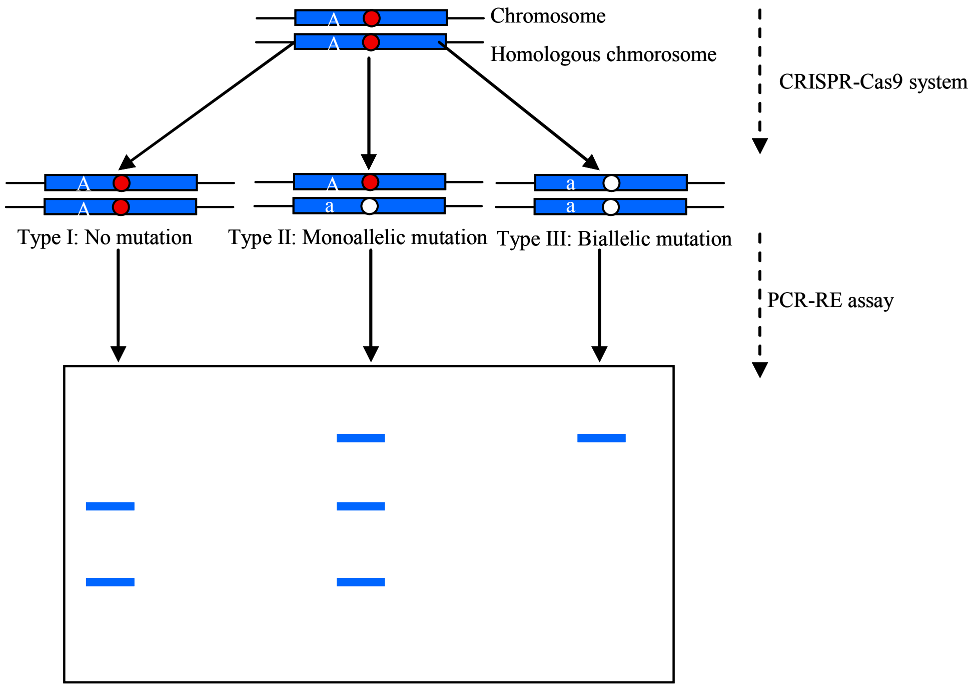

Figure S1. Schematic of the PCR-RE assay to detection of the mutants.

The target gene induced by the CRISPR-Cas9 system have three types in the hairy roots. Type I is the wild type with no mutation for the target gene. Type II is monoallelic mutation that one gene is mutation and the other allelic gene is no mutation. Type III is biallelic mutation which both of the two allelic genes are mutation. The gene is amplified using gene specific primers and then digested completely with the restriction enzyme (PCR-RE assay). When the gene mutation is induced by CRISPR/Cas9 system, the restriction enzyme site of the gene is destroyed. The product of PCR for this mutation cannot be digested by the enzyme. The results of PCR-RE assay for the non-mutation shows two digested bands. For the monoallelic mutation, the result is three bands with one undigested band from mutation gene, two digested bands from non-mutation allelic gene. For the biallelic mutation, both of the two allelic genes are mutation, the PCR-RE assay show only a single undigested band. A: wild-type gene; a: mutation gene; red circle: the restriction enzyme sites; white circle: no restriction enzyme sites.

a

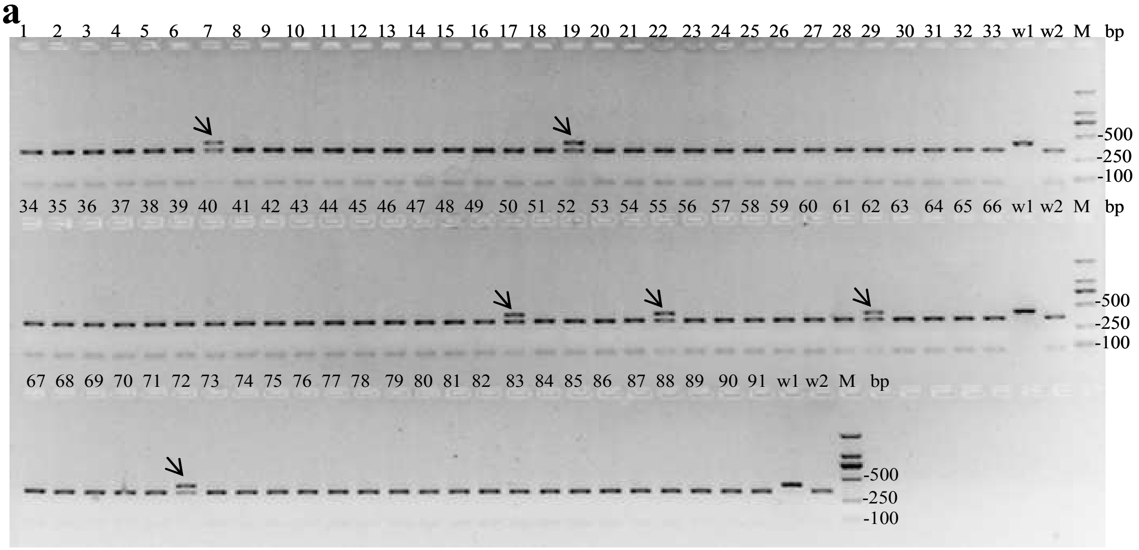

b

GTACTCTGCTGGCTGTGAAATTAACCAGCTGCAGTGGTCCGCCGCCCAGCCCGAT WT

monoallelic mutants ID

1 GTACTCTGCTGGCTGTGAAATTAACCAGCTGGCAGTGGTCCGCCGCCCAGCCCGAT +1(X1)

2 GTACTCTGCTGGCTGTGAAATTAACCAGCTGAAGTGGTCCGCCGCCCAGCCCGAT S1(X1)

3 GTACTCTGCTGGCTGTGAAATTAACCAGCTGACAGTGGTCCGCCGCCCAGCCCGAT +1(X1)

GTACTCTGCTGGCTGTGAAATTAACCAGCTGTCAGTGGTCCGCCGCCCAGCCCGAT +1(X2)

4 GTACTCTGCTGGCTGTGAAATTAACCAGCCGCAGTGGTCCGCCGCCCAGCCCGAT S1(X1)

5 GTACTCTGCTGGCTGTGAAATTAACCAGCTGGCAGTGGTCCGCCGCCCAGCCCGAT +1(X1)

6 GTACTCTGCTGGCTGTGAAATTAACCAGC---AGTGGTCCGCCGCCCAGCCCGAT -3(X2)

Figure S2. Targeted mutations in *Glyma06g14180* induced using the pCas9-AtU6-sgRNA vector.

(a) Detection of mutations by PCR-restriction enzyme (PCR-RE) in soybean hairy roots. Lanes 1–91: digested DNA of PCR products amplified from independent hairy root samples; The mutations are shown with arrow. w1 and w2: undigested and digested DNA of PCR products amplified from wild-type controls respectively; M: marker.

(b) Sequences of gene from the six independent monoallelic mutants. Wild-type sequences of the target gene was shown, with the protospacer-adjacent motif sequence highlighted in red. The change in the number of nucleotides is shown to the right of each sequence. +: insertion; D: deletion; S: substitution. Inserted and substituted nucleotides are shown in green. The number of clones for each mutant is given in brackets.

a

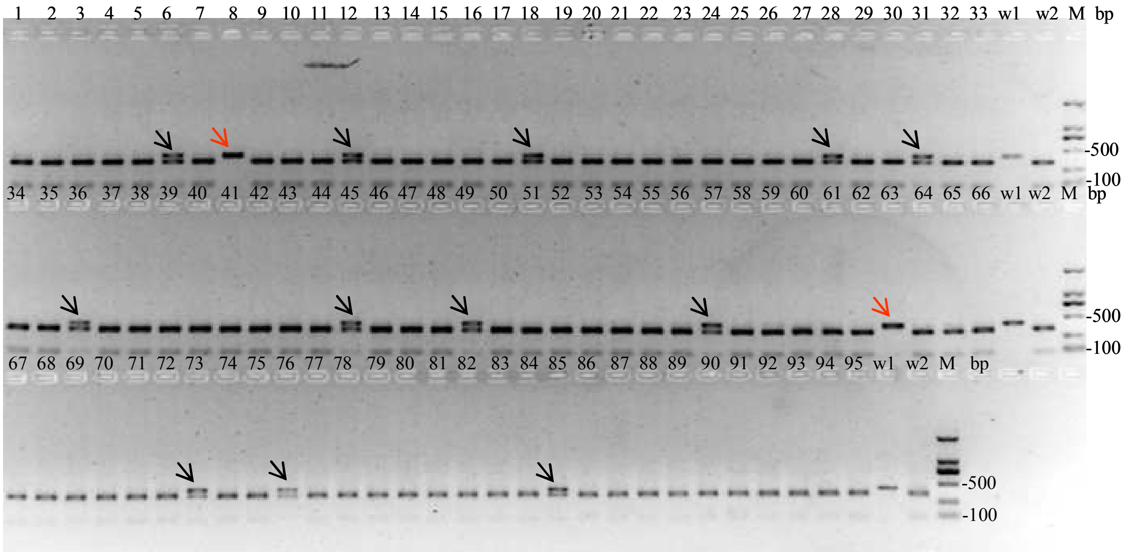

b

Glyma06g14180

GTACTCTGCTGGCTGTGAAATTAACCAGCTGCAGTGGTCCGCCGCCCAGCCCGAT WT

Off-target gene: *Glyma04g40610*

GTATTCTGCTGGCTGTGAAATTAACCAGCTGCAGTGGTCTGCCGTGCAGCCCGAT WT

monoallelic mutants

1 GTACTCTGCTGGCTATGAAATTAACCAGCTGACAGTGGTCCGCCGCCCAGCCCGAT +1(X2)

2 GTACTCTGCTGGCTGTGAAATTAACCAGCCGCAGTGGTCCGCCGCCCAGCCCGAT S1(X1)

3 GTACTCTGCTGGCTGTGAAATTAACCAGCTGTCAGTGGTCCGCCGCCCAGCCCGAT +1(X1)

Off-target

GTATTCTGCTGGCTGTGAAATTAACCAGCTGACAGTGGTCTGCCGTGCAGCCCGAT +1(X1)

GTATTCTGCTGGCTGTGAAATTAACCAGCTGGCAGTGGTCTGCCGTGCAGCCCGAT +1(X1)

4 GTACTCTGCTGGCTGTGAAATTAACCAGCTGGTCAGTGGTCCGCCGCCCAGCCCGAT +2(X1)

5 GTACTCTGCTGGCTGTGAAATTAACCAGCTGTCAGTGGTCCGCCGCCCAGCCCGAT +1(X1)

Off-target

GTATTCTGCTGGCTGTGAAATTAACCAGCTGTCAGTGGTCTGCCGTGCAGCCCGAT +1(X1)

6 GTACTCTGCTGGCTGTGAAATTAACCAGCTGTCAGTGGTCCGCCGCCCAGCCCGAT +1(X1)

Off-target

GTATTCTGCTGGCTGTGAAATTAACCAGCTGACAGTGGTCTGCCGTGCAGCCCGAT +1(X1)

GTATTCTGCTGGCTGTGAAATTAACCAGCTGGCAGTGGTCTGCCGTGCAGCCCGAT +1(X1)

7 GTACTCTGCTGGCTGTGAAATTAACCAGCTGACAGTGGTCCGCCGCCCAGCCCGAT +1(X1)

8 GTACTCTGCTGGCTGTGAAATTAACCA----CAGTGGTCCGCCGCCCAGCCCGAT -4(X2)

GTACTCTGCTGGCTGTGAAATTAACCAGCTGGCAGTGGTCCGCCGCCCAGCCCGAT +1(X1)

9 GTACTCTGCTGGCTGTGAAATTAACCAGCTGACAGTGGTCCGCCGCCCAGCCCGAT +1(X3)

GTACTCTGCTGGCTGTGAAATTAACCAGCTGTCAGTGGTCCGCCGCCCAGCCCGAT +1(X1)

10 GTACTCTGCTGGCTGTGAAATTAACCAGCTGACGGTGGTCCGCCGCCCAGCCCGAT +1,R1(X1)

GTACTCTGCTGGCTGTGAAATTAACCAGCTGTCAGTGGTCCGCCGCCCAGCCCGAT +1(X1)

Off-target

GTATTCTGCTGGCTGTGAAATTAACCAGCTGTCAGTGGTCTGCCGTGCAGCCCGAT +1(X1)

```
11  GTACTCTGCTGGCTGTGAAATTAACCAGCTGACAGTGGTCCGCCGCCCAGCCCGAT +1(X2)
    Off-target
    GTATTCTGCTGGCTGTGAAATTAACCAGCTGGCAGTGGTCTGCCGTGCAGCCCGAT +1(X2)
    GTATTCTGCTGGCTGTGAAATTAACCAGCTGGGCAGTGGTCTGCCGTGCAGCCCGAT +2(X2)
    GTATTCTGCTGGCTGTGAAATTAACCAGCTGGGTTTTGGTCTGCCGTGCAGCCCGAT +5,-3(X1)
12  GTACTCTGCTGGCTGTGAAATTAACCAGCTGGCAGTGGTCCGCCGCCCAGCCCGAT +1(X1)
    GTACTCTGCTGGCTGTGAAATTAACCAGCTGTCAGTGGTCCGCCGCCCAGCCCGAT +1(X1)
```

biallelic mutants

```
13  GTACTCTGCTGGCTGTGAAATTAACCAGCTGGCAGTGGTCCGCCGCCCAGCCCGAT +1(X1)
    GTACTCTGCTGGCTGTGAAATTAACCAGCTGTCAGTGGTCCGCCGCCCAGCCCGAT +1(X1)
    GTACTCTGCTGGCTGTGAAATTAACCAGCTGACAGTGGTCCGCCGCCCAGCCCGAT +1(X1)
    GTACTCTGCTGGCTGTGAAATTAACCAGCTG-AGTGGTCCGCCGCCCAGCCCGAT -1(X1)
    GTACTCTGCTGGCTGTGAAATTAACCAGCT---GTGGTCCGCCGCCCAGCCCGAT -3(X1)
14  GTACTCTGCTGGCTGTGAAATTAACCAGCTGTCAGTGGTCCGCCGCCCAGCCCGAT +1(X1)
    GTACTCTGCTGGCTGTGAAATTAACCAGCTGACAGTGGTCCGCCGCCCAGCCCGAT +1(X1)
    GTACTCTGCTGGCTGTGAAATTAACCAGCTGTACAGTGGTCCGCCGCCCAGCCCGAT +2(X3)
```

Figure S3. Targeted mutations in *Glyma06g14180* induced using the pCas9-GmU6-sgRNA vector.

(a) Detection of mutations by PCR-restriction enzyme (PCR-RE) in soybean hairy roots. Lanes 1–95: digested DNA of PCR products amplified from independent hairy root samples; The monoallelic and biallelic mutations are shown with black arrow and red arrow respectively. w1 and w2: undigested and digested DNA of PCR products amplified from wild-type controls respectively; M: marker.

(b) Sequences of gene from 14 independent mutants (12 monoallelic and 2 biallelic) induced by the pCas-GmU6-sgRNA vector. Sequences of *Glyma06g14180* and an off-target gene (*Glyma04g40610*) are shown at the top of the figure, with the protospacer-adjacent motif sequence highlighted in red. Nucleotides differing between *Glyma06g14180* and *Glyma04g40610* are shown in pink on the *Glyma04g40610* sequence. The off-targets were detected in the mutant 3,5,6,10 and 11. The change in the number of nucleotides is shown to the right of each sequence. +: insertion; D: deletion; S: substitution. Inserted and substituted nucleotides are shown in green. The number of clones for each mutant is given in brackets.

a

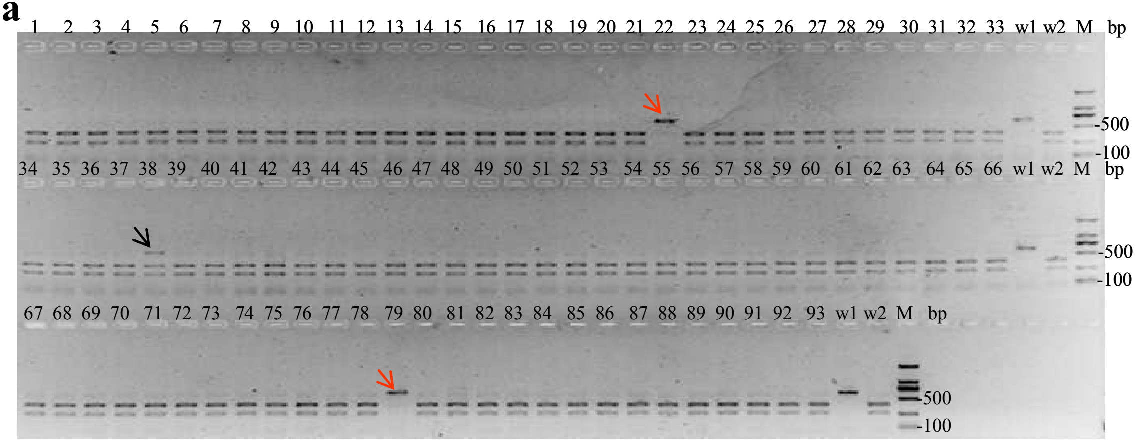

b

GAATGTTTCCCACGGGTTAAGGCAGTACATTCCAGAAGGTGGATCCCTGGTCAGACATATATAC WT

monoallelic mutant

1 TTTGAATGTTTCCCACGGGTTAAGGCAGTACATTCCAGAAGGTGGATTCCCTGGTCAGACATATATAC +1(X1)

TTTGAATGTTTCCCACGGGTTAAGGCAGTACATTCCAGAAGGTGG-TCCCTGGTCAGACATATATAC -1(X1)

biallelic mutants

2 GAATGTTTCCCACGGGTTAAGGCAGTACATTCCGGAAGGTGGA--TCTGGTCAGACATATATAC-2,S1(X1)

GAATGT—-------------------------------------CCCTGGTCAGACATATATAC -38(X3)

GAATGTTTCCCACGGGTTAAGGCAGTACATTCCAGAAGGTGGA--TCTGGTCAGACATATATAC -2(X2)

3 GAATGTTTCCCACGGGTTAAGGCAGTACATTCCAGAA------TCCCTGGTCAGACATATATAC -6(X4)

GAATGTTTCCCACGGGTTAAGGCAGTACATTCCAGAAGGTGGA------------CATATATAC -12(X1)

Figure S4. Targeted mutations in *Glyma08g02290* induced using the pCas9-AtU6-sgRNA vector.

(b) Detection of mutations by PCR-restriction enzyme (PCR-RE) in soybean hairy roots. Lanes 1–93: digested DNA of PCR products amplified from independent hairy root samples; The monoallelic and biallelic mutations are shown with black arrow and red arrow respectively. w1 and w2: undigested and digested DNA of PCR products amplified from wild-type controls respectively; M: marker.

(b) Sequences of gene from one monoallelic mutant and two biallelic mutants. Wild-type sequences of the target gene was shown, with the protospacer-adjacent motif sequence highlighted in red. The change in the number of nucleotides is shown to the right of each sequence. +: insertion; D: deletion; S: substitution. Inserted and substituted nucleotides are shown in green. The number of clones for each mutant is given in brackets.

a

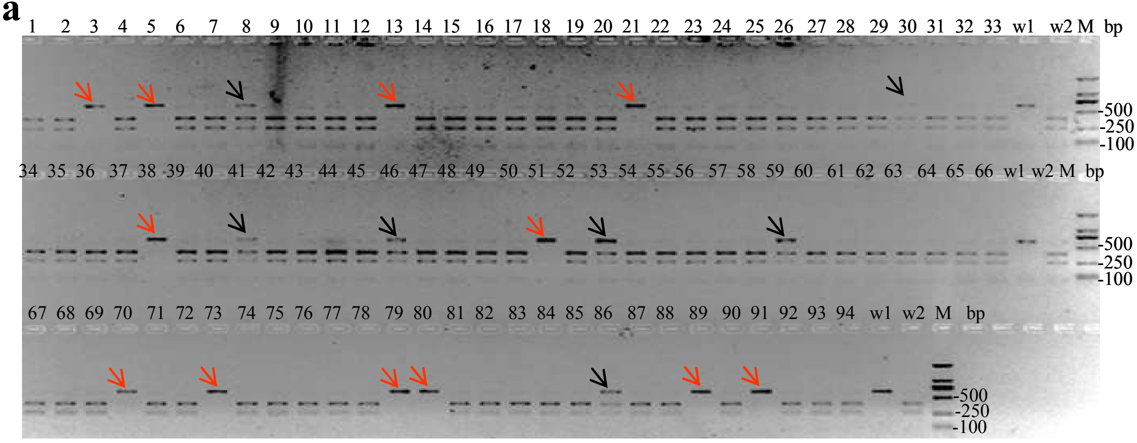

b

```
   GAATGTTTCCCACGGGTTAAGGCAGTACATTCCAGAAGGTGGATCCCTGGTCAGACATATATA WT
monoallelic mutant
1  GAATGTTTCCCACGGGTTAAGGCAGTACATTCCAGAAGGTGGATCCCCTGGTCAGACATATATA +1(X1)
2  GAATGTTTCCCACGGGTTAAGGCAGTACATTCCAGAA---------CTGGTCAAACATATATA -9(X1)
   GAATGTTTCCCACGGGTTAAGGCAGTACATTCCAGAA------TCCCTGGTCAGACATATATA -6(X1)
   GAATGTTTCCCACGGGTTAAGGCAGTACATTCCAGAAGGTGG--CCCTGGTCAGACATATATA -2(X1)
3  TTCCCACGGGTTAAGGCAGTACATTCCAGAAGGTGGATTCTTATGTAGTACACAGCATATATA +14,-8(X2)
4  GAATGTTTCCCACGGGTTAAGGCAGTACATTCCAGAAGGTGGA----TGGTCAGACATATATA -4(X2)
5  GAATGTTTCCCACGGGTTAAGGCAGTACATTCCAGAAGGTGGATTCCCTGGTCAGACATATGTA +1,S1(X3)
   GAATGTTTCCCACGGGTTAAGGCA---------------------------------TATATA -33(X1)
6  GAATGTTTCCCACGGGTTAAGGCAGTACATTCCAGAAGGTGGATTCCCTGGTCAGACATATATA +1(X1)
   GAATGTTTCACTGCCTTAACCCAGTTGGTTTC--------------------AGACATATAT +23,-43(X1)
7  GAATGTTTCCCACGGGTTAAGGCAGTACATTCCAGAAGGTGGATTCCCTGGTCAGACATATATA +1(X1)
   CCAT------(-33bp)------ACATTCCAGAAGGTGGCATTTGAATGTCTCCTGGTCAGA +20,-33 (X1)
biallelic mutants
1  GAATGTTTCCCACGGGTTAAGGCAGTACATTCCAGAATGTT--TCCCTGGTCAGACATATATA -2,S2(X2)
   GAATGTTTCCCACGGGTTAAGGCAGTACATTCCAGAAGGTGGAT--(-36bp)--TCTTATGATAA -36(X2)
2  GAATGTTTCCCACGGGTTAAGGCAGTACATTCCAGAAGGTGGATTCCCTGGTCAGACATATATA +1(X3)
   GAATGTTTCCCACGGGTTAAGGCAGTACATTCCAGAAGGTGGAT--CTGGTCAGACATATATA -2(X4)
3  GAATGTTTCCCACGGGTTAAGGCAGTACATTCCAGAAGGTGGATTCCCTGGTCAGACATATATA +1(X2)
   GAATGTTTCCCACGGGTTAAGGCAGTACATTCCAGAAGGTGGAT--CTGGTCAGACATATATA -2(X4)
4  GAATGTTTCCCACGGGTTAAGGCAGTACATTCCAGAAGGTGGATTCCCTGGTCAGACATATATA +1(X3)
   GAATGTTTCCCACGGGTTAAGGCTGTACATTCCAGAAGGTGGATTCCCTGGTCAGACATATATA +1,S1(X1)
   GAATGTTTCCCACGGGTTAAGGCAGTACATTCCA----------CCCTGGTCAGACATATATA -10(X1)
   GAATGTTTCCCACGGGTTAAGGCAGTACATTCC--------------TGGTCAGACATATATA -14(X1)
   GAATGTTTCCCACGGGTT-----(-33bp)-----CAGACATATATACCCGAGATAAACTGGA -33(X1)
5  GAATGTTTCCCACGGGTTAAGGCAGTACATTCCAGAAGGTGGAT--(-36bp)-TCTTATGATAAT -36(X4)
   TTCCCACGGGTTAAGGCAGTACATTCCAGAAGGTGGAT---TGG—-AGA—(-14bp)--ATAAACT -19(X1)
   TTCCCACGGGTTAAGGCAGTACATTCCAGAAG------------CCCTGGTCAAACATATATGC -12,S2(X1)
6  GAATGTTTCCCACGGGTTAAGGCAGTACATTCCAGAAGGTGGATTCCCTGGTCAGACATATGTA +1,S1(X4)
```

```
   GAATGTTTCCCACGGGTTAAGGCAGTACATTCCAGA--------CCCTGGTCAGACATATATA  -8(X4)
7  TCCAGAAGGTGGGAAACATTCAAATGCATGACCTGTTTATCCACCCCTGGTCAGACATATATA  +30(X3)
   GAATGTTTCCCACGGGTTAAGGCAGTACATTCC-------------CTGGTCAAACATATATA  -13(X2)
8  TTCCCACGGGTTAAGGCAGTACATTCCAGAAGGTGGAT---(-36bp)---TCTTATGATAAT  -36(X8)
9  GTTTCCCACGGGTTAAGGCAGTACATTCCAGAAGG--------(-57bp)-------TTGGTT  -57(X3)
   GAATGTTTCCCACGGGTTAAGGCAGTACATTCCAGA--------CCCTGGTCAGACATATATA  -8(X3)
10 GAATGTTTCCCACGGGTTAAGGCAGTACATTCCAG--------TCCCTGGTCAGACATATATA  -8(X3)
   GAATGTTTCCCACGGGTTAAGGCAGTACAT----(-32bp)--- ACCCGAGATAAACTGGATT  -32(X3)
11 GAATGTTTCCCACGGGTTAAGGCAGTACATTCCAGAAGGTGG----CTGGTCAGACATATATA  -4(X2)
   GAATGTTTCCCACGGGTTAAGGCAGTACATTCCAGAAGGTGGATTCCCTGGTCAGACATATATA  +1(X3)
   GAATGTTTCCCACGGGTTAAGGCAGTACATTCCAGAAGGT-----------CAGACATATATA  -11(X1)
   GAATGTTTCCCACGGGTTAAGGCAGTACATTCCAGAAGGTGGATCCCTG-TCAGACATATATA  -1(X1)
12 GAATGTTTCCCACGGGTTAAGGCAGTACATTCCAGAAGGTGGATTCTTAACCCCTGGTCAGACATATA +7(X3)
   GAATGTTTCCCACGGGTTAAGGCAGTACATTCCAGAAGGTGGACCCCTGGTCAGACATATATA  R1(X2)
   GAATGTTTCCCACGGGTTAAGGCAGTACATTCCAGAAGGTGGATTCCCTGGTCAGACATATGTA  +1,S1(X1)
```

Figure S5. Targeted mutations in *Glyma08g02290* induced using the pCas9-GmU6-sgRNA vector.

(a) Detection of mutations by PCR-restriction enzyme (PCR-RE) in soybean hairy roots. Lanes 1–94: digested DNA of PCR products amplified from independent hairy root samples; The monoallelic and biallelic mutations are shown with black arrow and red arrow respectively. w1 and w2: undigested and digested DNA of PCR products amplified from wild-type controls respectively; M: marker.

(b) Sequences of gene from 19 independent mutants (12 monoallelic and 7 biallelic mutants) induced by the pCas-GmU6-sgRNA vector. Sequences of *Glyma06g14180* is shown with the protospacer-adjacent motif sequence highlighted in red. The change in the number of nucleotides is shown to the right of each sequence. +: insertion; D: deletion; S: substitution. Inserted and substituted nucleotides are shown in green. The number of clones for each mutant is given in brackets.

a

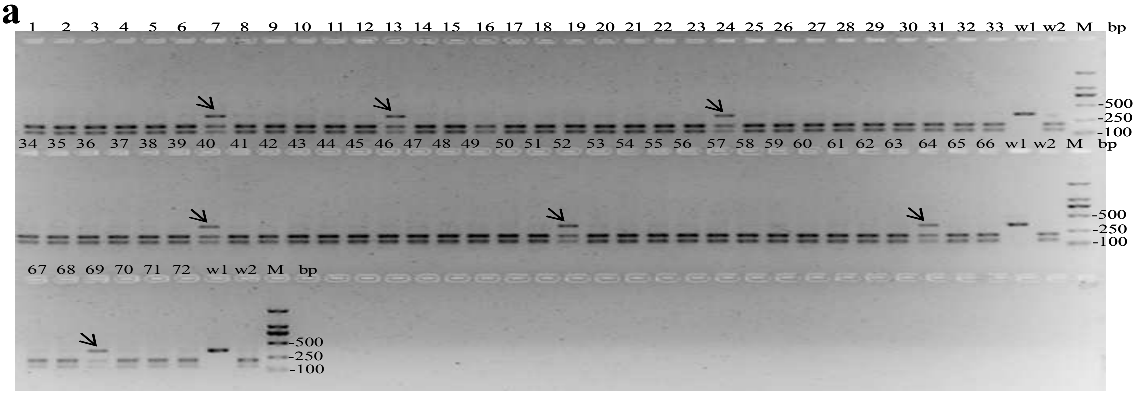

b

Glyma12g37050

GTGGTCATTACAACAGCCACAGTTCTTGAATGAATTCTAAAGGTCCATAAATTAATTAGATGT WT

Off-target gene: *Glyma09g00490*

GTGGTCATTACAACAGCCACAGTTCTTGAATGAATTCTAAATGTCCATAAATTAATTAGATGT WT

1 GTGGTCATTACAACAGCCACAGTTCTTGAATGAA-----AAGGTCCATAAATTAATTAGATGT -5(X1)

2 GTGGTCATTACAACAGCCACAGTTCTTGAATGAATT--AAAGGTCCATAAATTAATTAGATGT -2(X1)

3 GTGGTCATTACAACAGCCACAGTTCTTGAATGAATT-TAAAGGTCCATAAATTAATTAGATGT -1(X1)

GTGGTCATTACAACAGCCACAGTTCTTGAATGAA---TAAAGGTCCATAAATTAATTAGATGT -3(X1)

Off-target

GTGGTCATTACAACAGCCACAGTTCTTGAATGAGTTCTAAATGTCCATAAATTAATTAGATGT S1(X1)

4 GTGGTCATTACAACAGCCACAGTTCTTGAATGAAT---AAAGGTCCATAAATTAATTAGATGT -3(X1)

5 GTGGTCATTACAACAGCCACAGTTCTTGAATGAAT---AAAGGTCCATAAATTAATTAGATGT -3(X2)

6 GTGGTCATTACAACAGCCACAGTTCTTGAATG-----TAAAGGTCCATAAATTAATTAGATGT -5(X1)

7 GTGGTCATTACAACAGCCACAGTTCTTGAATGA----TAAAGGTCCATAAATTAATTAGATGT -4(X1)

GTGGTCATTACAACAGCCACAGTTCTTGAATGAA---TAAAGGTCCATAAATTAATTAGATGT -3(X1)

Figure S6. Target mutations in *Glyma12g37050* induced using the pCas9-AtU6-sgRNA vector.

(a) Detection of mutations by PCR-restriction enzyme (PCR-RE) in soybean hairy roots. Lanes 1–72: digested DNA of PCR products amplified from independent hairy root samples; The mutations are shown with arrow. w1 and w2: undigested and digested DNA of PCR products amplified from wild-type controls respectively; M: marker.

(b) Sequences of gene from 7 independent mutants induced by the pCas9-AtU6-sgRNA vector. Sequences of *Glyma12g37050* and an off-target gene (*Glyma09g00490*) are shown with the protospacer-adjacent motif sequence highlighted in red. Nucleotides differing between *Glyma12g37050* and *Glyma09g00490* are shown in pink on the *Glyma09g00490* sequence. The off-target was detected in the mutant 3. The change in the number of nucleotides is shown to the right of each sequence. +: insertion; D: deletion; S: substitution. Substituted nucleotides are shown in green. The number of clones for each mutant is given in brackets.

a

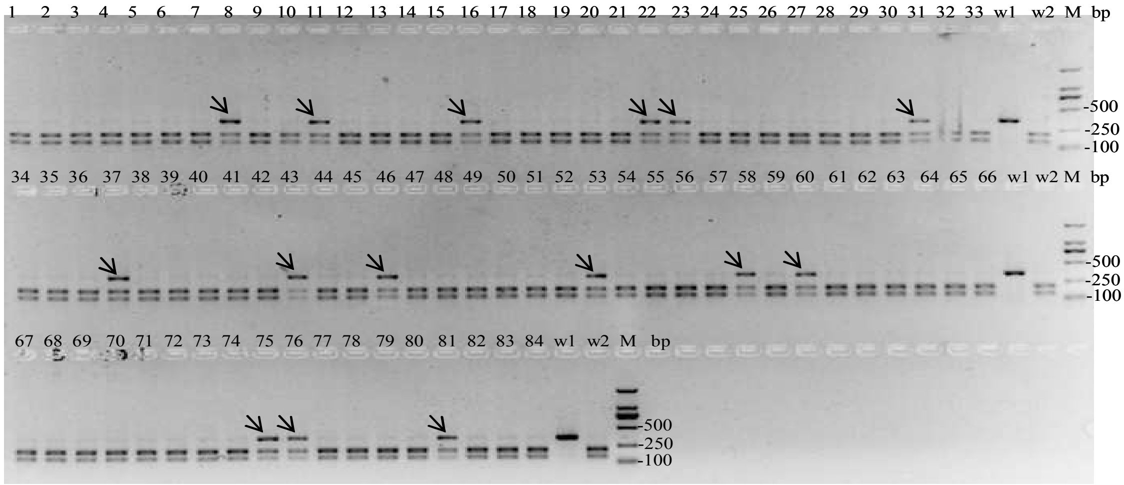

b

Glyma12g37050

GTGGTCATTACAACAGCCACAGTTCTTGAATGAATTCTAAAGGTCCATAAATTAATTAGATGT WT

Off-target gene: *Glyma09g00490*

GTGGTCATTACAACAGCCACAGTTCTTGAATGAATTCTAAATGTCCATAAATTAATTAGATGT WT

1 GTGGTCATTACAACAGCCACAGTTCTTGAATGAA-----AAGGTCCATAAATTAATTAGATGT -5(X1)

2 GTGGTCATTACAACAGCCACAGTTCTTGAATGAATT--AAAGGTCCATAAATTAATTAGATGT -2(X1)

3 GTGGTCATTACAACAGCCACAGTTCTTGAATGAATTTTTAAAGGTCCATAAATTAATTAGATGT S1,+1(X1)

GTGGTCATTACAACAGCCACAGTTCTTGAATGAAT---AAAGGTCCATAAATTAATTAGATGT -3(X1)

GTGGTCATTACAACAGCCACAGTTCTTGAATGAATT--AAAGGTCCATAAATTAATTAGATGT -2(X1)

4 GTGGTCATTACAACAGCCACAGTTCTTGAATGA----TAAAGGTCCATAAATTAATTAGATGT -4(X1)

5 GTGGTCATTACAACAGCCACAGTTCTTGAATGA----TAAAGGTCCATAAATTAATTAGATGT -4(X1)

GTGGTCATTACAACAGCCACAGTTCTTGAGTGA----TAAAGGTCCATAAATTAATTAGATGT S1,-4(X1)

6 GTGGTCATTACAACAGCCACAGTTCTTGAATGA----TAAAGGTCCATAAATTAATTAGATGT -4(X2)

7 GTGGTCATTACAACAGCCACAGTTCTTGAATGAATT----AGGTCCATAAATTAATTAGATGT -4(X1)

GTGGTCATTACAACAGCCACA------------------AAGGTCCATAAATTAATTAGATGT -18(X1)

8 GTGGTCATTACAACAGCCACAGTTCTTGAATGAAT-----AGGTCCATAAATTAATTAGATGT -5(X1)

9 GTGGTCATTACAACAGCCACAGTTCTT-----------AAAGGTCCATAAATTAATTAGATGT -11(X1)

10 GTGGTCATTACAACAGCCACAGTTCTTGAATGA----TAAAGGTCCATAAATTAATTAGATGT -4(X1)

GTGGTCATTACGACAGCCACAGTTCTAATTTGATGGAGCAATGGCCATTACAACAGCCACATCTAATTAATTT

ATGTGGAGCAATGGTCATTACAACAGCCACATCTAATTTGACACAGTTAAAGGTCCATA R1,-11,+94(X1)

Off-target

GTGGTCATTACAACAGCCACAGTTCTTGAATGAACTCTAAATGTCCATAAATTAATTAG S1(X1)

11 GTGGTCATTACAACAGCCACAGTTCTTGAATGAATT--AAAGGTCCATAAATTAATTAGATGT -2(X1)

GTGGTCATTACAACAGCCACAGTTCTTGAAT-------AAAGGTCCATAAATTAATTAGATGT -7(X1)

12 GTGGTCATTACAACAGCCACAGTTCTTGAAT-----CTAAAGGTCCATAAATTAATTAGATGT -5(X1)

13 GTGGTCATTACAACAGCCACAGTTCTTGAATGAA-----AAGGTCCATAAATTAATTAGATGT -5(X1)

GTGGTCATTACAACAGCCACAGTTCTTGAATGAA-----AAGGTCCATAAACTAATTAGATGT -5,S1(X1)

14 GTGGTCATTACAACAGCCACAGTTCTTGAATGAAT---AAAGGTCCATAAATTGATTAGATGT -3,S1(X1)

15 GTGGTCATTACAACAGCCACAGTTCTTGAATGA---CTAAAGGTCCATAAATTAATTAGATGT -3(X1)

GTGGTCATTACAACAGCCACAGTTCTTGAAT-------AAAGGTCCATAAATTAATTAGATGT -7

Figure S7. Target mutations in *Glyma12g37050* induced using the pCas9-GmU6-sgRNA vector.

(a) Detection of mutations by PCR-restriction enzyme (PCR-RE) in soybean hairy roots. Lanes 1–84: digested DNA of PCR products amplified from independent hairy root samples; The mutations are shown with arrow. w1 and w2: undigested and digested DNA of PCR products amplified from wild-type controls respectively; M: marker.

(b) Sequences of gene from 15 independent mutants induced by the pCas9-GmU6-sgRNA vector. Sequences of *Glyma12g37050* and an off-target gene (*Glyma09g00490*) are shown with the protospacer-adjacent motif sequence highlighted in red. Nucleotides differing between *Glyma12g37050* and *Glyma09g00490* are shown in pink on the *Glyma09g00490* sequence. The off-target was detected in the mutant 10. The change in the number of nucleotides is shown to the right of each sequence. +: insertion; D: deletion; S: substitution. Inserted and substituted nucleotides are shown in green. The number of clones for each mutant is given in brackets.

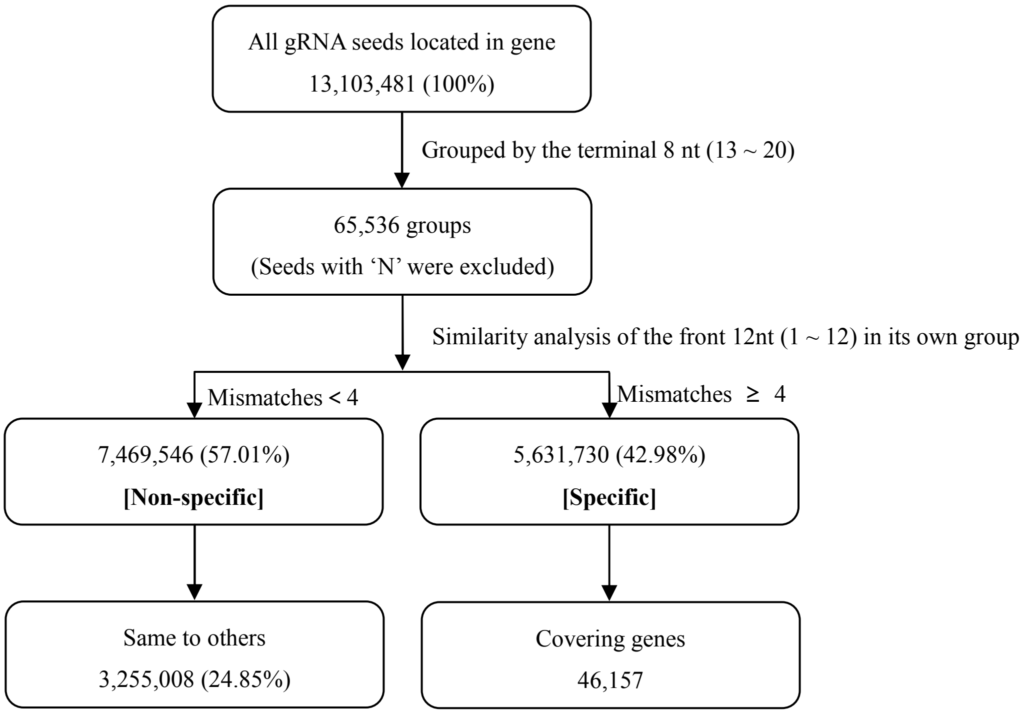

Supplementary Figure S8. Strategy used for genome-wide prediction of synthetic guide RNA seeds in soybean genes.

Pipeline of specific sgRNA seeds analysis in the soybean genome. Firstly, 20 nt long sgRNA seeds were excluded from both strands of chromosome sequences. Second, these sgRNA seeds were grouped according to the identity of the terminal 8 nt at their 3'-end. Third, similarity alignment of the front 12 nt was performed in its own group to identify mismatch numbers. Four mismatches is deems as threshold to divide specific and non-specific sgRNA seeds. Last, totally same sgRNA seeds in non-specific class and covering gene number of specific category were also indicated.

GmU6-10-sgRNA

gaattcgagctcAAAATAAATGGTAAAATGTCAAATCAAAACTAGGCTGCAGTATGCAGAGCA
EcoR I *Sac* I

GAGTCATGATGATACTACTTACTACACCGATTCTTGTGTGCAGAAAAATATGTTAAAATA

ATTGAATCTTTCTCTAGCCAAATTTGACAACAATGTACACCGTTCATATTGAGAGACGAT

GCTTCTTGTTTGCTTTCGGTGGAAGCTGCATATACTCAACATTACTCCTTCAGCGAGTTT

TCCAACTGAGTCCCACATTGCCCAGACCTAACACGGTATTCTTGTTTATAATGAAATGT

GCCACCACATGGATTGAgagaccAAggtctcAGTTTTAGAGCTAGAAATAGCAAGTTAAAATA
Bsa I *Bsa* I

AGGCTAGTCCGTTATCAACTTGAAAAAGTGGCACCGAGTCGGTGCTTTTTTTTaagctt
Hind III

Supplementary Figure S9. DNA sequence of GmU6-10-sgRNA. Blue: the sequence of soybean U6-10 promoter; Red: the sequence of sgRNA; The digested Enzymes are show with lowercase letter.

AtU6-26-sgRNA

gaattcgagctcGTTGAACAACGGAAACTCGACTTGCCTTCCGCACAATACATCATTTCTTCTT
EcoR I *Sac* I

AGCTTTTTTTCTTCTTCTTCGTTCATACAGTTTTTTTTTGTTTATCAGCTTACATTTTCTTG

AACCGTAGCTTTCGTTTTCTTCTTTTTAACTTTCCATTCGGAGTTTTTGTATCTTGTTTCA

TAGTTTGTCCCAGGATTAGAATGATTAGGCATCGAACCTTCAAGAATTTGATTGAATAA

AACATCTTCATTCTTAAGATATGAAGATAATCTTCAAAAGGCCCCTGGGAATCTGAAAG

AAGAGAAGCAGGCCCATTTATATGGGAAAGAACAATAGTATTTCTTATATAGGCCCATTT

AAGTTGAAAACAATCTTCAAAAGTCCCACATCGCTTAGATAAGAAAACGAAGCTGAGT

TTATATACAGCTAGAGTCGAAGTAGTGATTGAgagaccAAggtctcAGTTTTAGAGCTAGAAA
Bsa I *Bsa* I

TAGCAAGTTAAAATAAGGCTAGTCCGTTATCAACTTGAAAAAGTGGCACCGAGTCGGT

GCTTTTTTTTaagctt
Hind III

Supplementary Figure S10. DNA sequence of AtU6-26-sgRNA. Blue: the sequence of *Arabidopsis* U6-26 promoter; Red: the sequence of sgRNA; The digested Enzymes are show with lowercase letter.

Supplementary Table S1. Chromosomal locations of soybean *U6* genes.

Gene	Chromosome	Strand	Location
U6-1	Gm04	+	4,045,962–4,046,064
U6-2	Gm06	+	3,834,623–3,834,725
U6-3	Gm09	+	1,012,810–1,012,912
U6-4	Gm13	-	29,940,513–29,940,615
U6-5	Gm15	+	9,241,899–9,242,001
U6-6	Gm15	-	49,397,436–49,397,538
U6-7	Gm16	+	5,025,475–5,025,577
U6-8	Gm16	+	5,031,456–5,031,556
U6-9	Gm19	-	33,969,480–33,969,582
U6-10	Gm19	+	34,369,044–34,369,146
U6-11	Gm19	+	34,410,271–34,410,373

Supplementary Table S2. Target sequences of three genes in soybean and oligonucleotides used to expressed sgRNA in the vectors.

Target gene	Target site sequences	Oligo forward (5'-3')	Oligo reverse (5'-3')	Enzyme	Gene annotation
Glyma06g14180	GTGAAATTAACCAGCTGCAGTGG	attg GTGAAATTAACCAGCTGCAG	aaac CTGCAGCTGGTTAATTTCAC	*Pst* I	WD domain containing protein
Glyma08g02290	CATTCCAGAAGGTGGATCCCTGG	attg CATTCCAGAAGGTGGATCCC	aaac GGGATCCACCTTCTGGAATG	*Bam*H I	K+ potassium transporter
Glyma12g37050	AGTTCTTGAATGAATTCTAAAGG	attg AGTTCTTGAATGAATTCTAA	aaac TTAGAATTCATTCAAGAACT	*Eco*R I	GAF domain containing protein

The restriction enzyme sites are showed in green color. The PAMs are highlighted with red.

Supplementary Table S3. List of primers in this study

Usage	Primer name	Sequence (5'-3')
Cloning	Cas9-F	CATGccatggCCCCAAAGAAGAAGCGC
	Cas9-R	TCAATCGCCGCCGAGTTGTGA
Mutation detection	Glyma06g14180-F	GGAGCACTCCACCATCATCTAC
	Glyma06g14180-R	GTTCTGACCTCAAACCTTCAAA
	Glyma08g02290-F	TTAACTTCTCATGCTGGTGTG
	Glyma08g02290-R	ACGTGTGTTCTGTTTTCTGGT
	Glyma12g37050-F	CATGTCGACCAGTTTCTTCTT
	Glyma12g37050-R	TGCTTATTTCTCAATCCCTTT
